# The US Influenza Hospitalization Surveillance Network 

**DOI:** 10.3201/eid2109.141912

**Published:** 2015-09

**Authors:** Sandra S. Chaves, Ruth Lynfield, Mary Lou Lindegren, Joseph Bresee, Lyn Finelli

**Affiliations:** Centers for Disease Control and Prevention, Atlanta, Georgia, USA (S.S. Chaves, J. Bresee, L. Finelli);; Minnesota Department of Health, St. Paul, Minnesota, USA (R. Lynfield);; Vanderbilt University School of Medicine, Nashville, Tennessee, USA (M.L. Lindegren)

**Keywords:** Influenza, influenza hospitalizations, population-based surveillance, Emerging Infections Program, EIP, viruses, US Influenza Hospitalization Surveillance Network, FluSurv-NET

## Abstract

In 2003, surveillance for influenza in hospitalized persons was added to the Centers for Disease Control and Prevention Emerging Infections Program network. This surveillance enabled monitoring of the severity of influenza seasons and provided a platform for addressing priority questions associated with influenza. For enhanced surveillance capacity during the 2009 influenza pandemic, new sites were added to this platform. The combined surveillance platform is called the Influenza Hospitalization Surveillance Network (FluSurv-NET). FluSurv-NET has helped to determine the risk for influenza-associated illness in various segments of the US population, define the severity of influenza seasons and the 2009 pandemic, and guide recommendations for treatment and vaccination programs.

## Abstract 2

This network has helped determine risk, define outbreak severity, and guide recommendations for treatment and vaccination programs.

Every year, influenza virus circulates worldwide, causing substantial illness and death and leading to considerable economic losses (*1*). From time to time, new strains of influenza A viruses emerge and cause a global pandemic with devastating consequences (*2*–*4*). Therefore, influenza surveillance programs are crucial for monitoring the timing and severity of seasonal influenza, which virus strains are circulating in a community, and changes in the epidemiology or risk associated with influenza virus infection. These data can be used to plan for vaccine strain selection, to alert the medical community and public health officials about the intensity and magnitude of an epidemic, and to evaluate the effects of intervention programs. In the event of an influenza pandemic, surveillance programs are essential for guiding response efforts and assisting with resource prioritization.

In response to the 2003–04 influenza season, which was relatively severe and caused a large number of deaths among healthy children, the Centers for Disease Control and Prevention (CDC) and 10 state health departments initiated a population-based surveillance system for laboratory-confirmed influenza in hospitalized children <18 years of age (*5*). Surveillance for influenza-associated hospitalizations among children proved to have useful public health implications, such as informing influenza vaccine recommendations over the years (*6*,*7*). Two years later, in 2005, the system was expanded to include surveillance for influenza hospitalizations among adults and was named the Influenza Hospitalization Surveillance Network (FluSurv-NET). FluSurv-NET data have helped determine the risk for illness in various segments of the population, document the severity of specific influenza seasons, and guide recommendations for treatment and vaccination programs. We describe FluSurv-NET, discuss how the system has generated data for public health action, and describe achievements and new directions for improving the system.

## Key Components of FluSurv-NET

The CDC Emerging Infections Program (EIP) platform is the cornerstone of FluSurv-NET and since the 2003–04 influenza season has conducted ongoing, population-based surveillance for children hospitalized with influenza (*5*). Surveillance for adults hospitalized with influenza was added to the EIP platform during the 2005–06 season (*8*). EIP sites include selected counties in California, Colorado, Connecticut, Georgia, Maryland, Minnesota, New Mexico, New York, Oregon, and Tennessee. To enhance surveillance, using the same EIP hospitalization surveillance protocol and making the system more geographically representative, new sites were added during the 2009 influenza pandemic. FluSurv-NET currently comprises the previously listed 10 EIP sites plus Michigan, Ohio, and Utah. The network encompasses 267 acute care hospitals and laboratories and has a total catchment area of >27 million persons (≈9% of the US population). Distribution of age, sex, race/ethnicity, and health indicators (e.g., population density and percentage of persons at or below poverty level) for persons in the FluSurv-NET catchment area is similar to that for persons throughout the nation.

FluSurv-NET monitors community-acquired, laboratory-confirmed influenza-related hospitalizations, defined as hospitalization of persons residing in the surveillance area at 1 of the catchment area hospitals <14 days after or <3 days before a positive influenza test result during October 1–April 31 each year. Because of the long-standing relationship between public health officials, academic centers and private hospitals, and laboratories at each participating site, and because of the feasibility of channeling resources to external partners through cooperative agreements, surveillance can be extended beyond seasonal periods and special projects can be implemented in the FluSurv-NET platform at very short notice.

**Figure 1:**
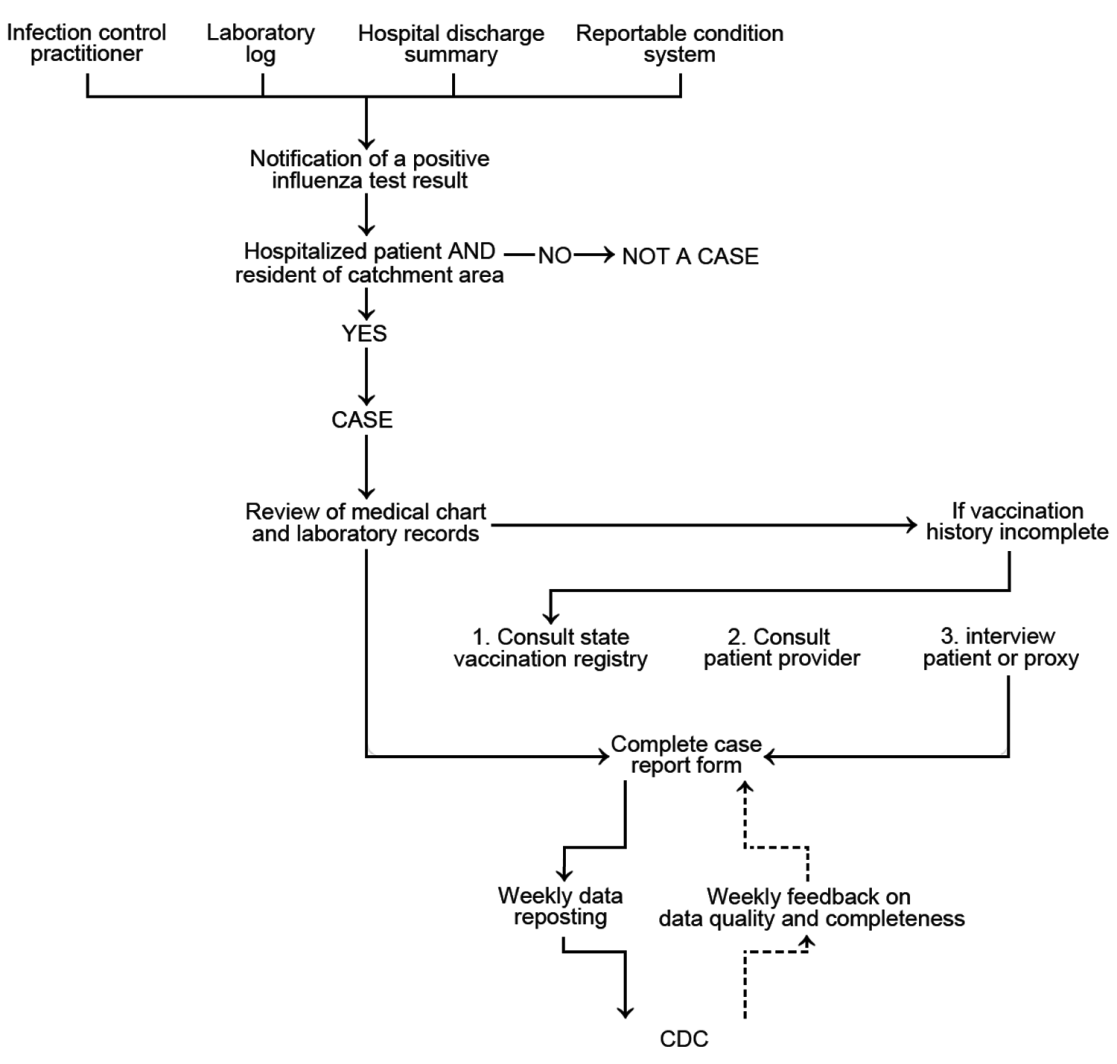
Population-based influenza hospitalization surveillance case ascertainment and review process, Influenza Hospitalization Surveillance Network, United States. Core data transmitted weekly to the Centers for Disease Control and Prevention (CDC) are patient identification number, surveillance site, hospital admission date, patient’s date of birth, type of influenza test, and type of influenza virus. Case finding and chart reviews are done manually.

Surveillance officers (either at an academic center or public health department) are hired and trained to collect information about patients hospitalized with influenza. A laboratory-confirmed influenza case is identified from laboratory logs of diagnostic testing for influenza, patient medical records, infection control practitioners’ databases/logs, or weekly calls to catchment-area hospitals. Cases can also be identified by reportable conditions databases at sites where influenza hospitalization is a reportable public health condition (Figure 1). Each positive influenza test result is investigated as to whether it represents a hospitalization event; this process helps avoid data entry duplication. Laboratory testing for influenza is ordered at the discretion of clinicians providing care. Laboratory confirmation is defined by a positive result from viral culture, direct or indirect fluorescent antibody staining, rapid antigen testing, or real-time reverse transcription PCR (RT-PCR). Hospitals are encouraged to send specimens to the public health department laboratory for RT-PCR confirmation and additional virus characterization, including virus subtyping.

Information about patient demographic characteristics and clinical course of illness during hospitalization is collected for each laboratory-confirmed influenza case through review of medical records by use of a standard form (Technical Appendix). Clinical data collected depict presence of underlying chronic medical conditions, influenza treatment and vaccination, clinical outcomes during hospitalization (including admission to an intensive care unit, need for mechanical ventilation, and death), and hospital discharge diagnoses. Influenza vaccination status is obtained through review of medical records and vaccination registries, primary care provider, or interview of patients or their proxies.

FluSurv-NET data collection was determined by CDC to be public health surveillance and therefore not subject to CDC Institutional Review Board approval for human research protections. Nonetheless, each participating site determines the need to submit the study to its state and local institutional review boards.

### Surveillance 

**Figure 2:**
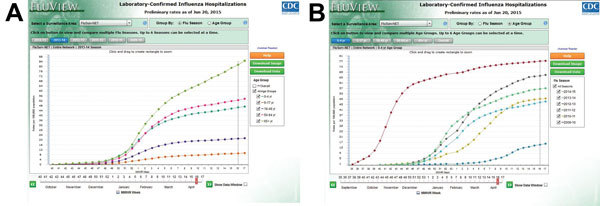
Screenshot of FluView web-based interaction application showing cumulative laboratory-confirmed influenza-associated hospitalizations per 100,000 population, United States. A) Age-specific rates by age groups; B) rates within specific age group, by influenza season. MMWR week defined at http://wwwn.cdc.gov/nndss/document/MMWR_Week_overview.pdf. Data from http://gis.cdc.gov/GRASP/Fluview/FluHospRates.html.

**Figure 3:**
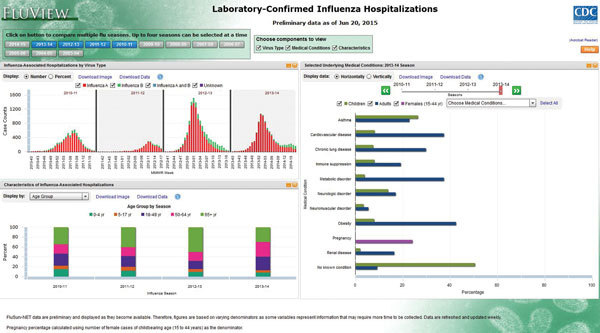
Screenshot of FluView web-based interaction application showing characteristics of hospitalized patients with laboratory-confirmed influenza in the United States by virus type; selected demographic characteristics, by influenza season; and prevalence of underlying medical conditions in children and adults**.** Data from http://gis.cdc.gov/grasp/fluview/FluHospChars.html.

During an influenza season, data collected through FluSurv-NET are reported weekly to the CDC Influenza Division (required core variables are surveillance site; hospital admission date; patient’s date of birth; type of influenza test; and, if available, type of influenza virus). There is a median lag time of 7 days (range 2–10 days) between date of a positive influenza test result and reporting of core variables to CDC. The primary product of FluSurv-NET is age-specific rates of laboratory-confirmed influenza-associated hospitalizations in the United States, which are calculated by using population denominators from the most recent census data available for each surveillance county catchment area. These rates are made available weekly in a web-based interactive application (available at http://gis.cdc.gov/GRASP/Fluview/FluHospRates.html and http://gis.cdc.gov/grasp/fluview/FluHospChars.html), which can also provide visualization of much of the influenza data collected and analyzed by CDC. This interactive application allows for analyses and visualization of customized data and comparisons across influenza seasons, regions, age groups, and selected patient demographics and clinical characteristics (Figures 2, 3). Selective clinical characteristics posted in the interactive application probably reflect 25%–50% of reported cases with complete clinical information at any given time during the season. Full clinical data for all hospitalized patients are often available after the end of the season, when chart reviews are finalized and providers, patients, or both have been interviewed regarding influenza vaccination.

Useful features of this system include near real-time information about the current influenza season and comparison of rates from previous seasons and by age groups. Data reported from FluSurv-NET are posted on a CDC website in a weekly influenza surveillance report prepared by the Influenza Division and called FluView (http://www.cdc.gov/flu/weekly/). Data posted in FluView are used to respond to media calls, address public information needs, and provide national-level information to local and state health departments for use in interpreting and communicating information about the influenza season in their own jurisdiction.

During the 2009 influenza pandemic, FluSurv-NET provided a crucial source of data for policy and decision making. Demographic and risk factor data from FluSurv-NET were used to develop vaccination prioritization recommendations for monovalent influenza A(H1N1)pdm09 virus vaccine distribution and administration early in the pandemic when the vaccine was in short supply. FluSurv-NET data were also used to demonstrate that persons in some age groups, with conditions such as pregnancy (*9*), and in some racial/ethnic groups were at higher risk for severe health outcomes associated with A(H1N1)pdm09 infection (*10*). In addition, these data contributed to the development of antiviral medication prioritization recommendations in anticipation of antiviral medication shortages.

Hospitalization rate data were used on a weekly basis to brief the CDC director and senior leadership at the Department of Health and Human Services about the severity and magnitude of the pandemic. Furthermore, data from this system were modeled to estimate national disease burden and became a monthly public benchmark for estimating how hard the pandemic was hitting the country. These data confirmed that morbid obesity was a new risk factor for influenza-related complications (*11*) and served as a reminder of the severe toll that influenza can take on persons with concurrent medical conditions, such as children with underlying neurologic disabilities (*12*).

### Addressing Critical Public Health Questions

Historically, estimates of the burden of influenza disease have relied on modeling excess influenza-associated hospitalizations by using national hospital discharge data (*13*,*14*). Therefore, results were available retrospectively only, after a 2–3 year delay. This delay precluded the use of burden estimates and season severity assessments to guide hospital resource allocations, control and preventive interventions, and public messaging. However, since the 2009 influenza pandemic, FluSurv-NET has provided a platform for contemporaneous timely assessments of seasonal severity and influenza disease burden estimates (*15*,*16*). The FluSurv-NET disease burden model uses probabilistic models to account for age-specific influenza testing practices and case underreporting and extrapolates data from FluSurv-NET sites to arrive at national estimates. Most recently, it was estimated that from the 2010–11 season through the 2012–13 season, 114,192–624,435 hospitalizations, 18,491–95,390 intensive care admissions, and 4,915–27,174 deaths occurred per year (*16*).

**Figure 4:**
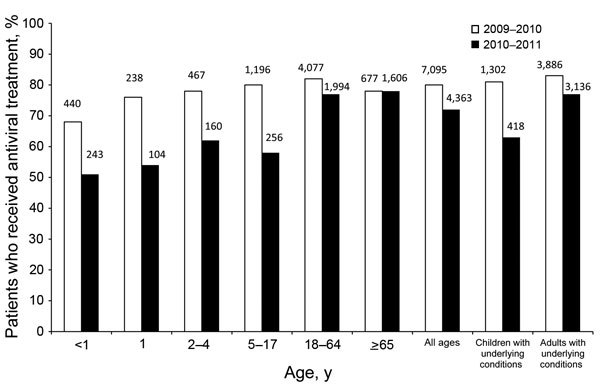
Percentages of children and adults hospitalized with laboratory-confirmed influenza virus infection who received influenza antiviral treatment, during 2009–10 (total hospitalized patients = 8,866) and 2010–11 (total hospitalized patients = 6,040), United States. Numbers above bars denote numbers of patients who received influenza antiviral treatment. p<0.01 for all age groups and categories except for the age group >65 years (*18*). Data from FluSurv-NET.

FluSurv-NET data have also been used to evaluate prescription of antiviral medication for hospitalized persons in the United States (*17*,*18*). Influenza antiviral medications are recommended for all hospitalized persons with suspected or confirmed cases of influenza. Despite the increased use of antiviral medications observed during the 2009 influenza pandemic, use of these medications declined substantially after the pandemic, especially among children (*18*) (Figure 4). These results were used to educate clinicians and improve messaging about the use of antiviral medications as a way to accelerate recovery and reduce influenza-associated complications, especially among hospitalized patients.

During the 2009 influenza pandemic, the FluSurv-NET platform was used to evaluate the effectiveness of the monovalent influenza A(H1N1)pdm09 vaccine. Effectiveness of the vaccine for preventing hospitalizations was estimated to be 50% (95% CI 13%–71%) (*19*); through the vaccine effectiveness study, FluSurv-NET contributed substantially to the evaluation of the A(H1N1)pdm09 prevention and control plan. Concerns were also expressed about the monovalent influenza A(H1N1)pdm09 vaccine being associated with Guillain-Barré syndrome (GBS); therefore, another contribution of the FluSurv-NET system during the 2009 influenza pandemic was completion of studies demonstrating a very small association of the monovalent influenza A(H1N1)pdm09 vaccine with risk for GBS (*20*,*21*). The attributable risk was similar to that previously estimated for seasonal influenza vaccine (≈1–2 cases/1 million doses administered), suggesting a low risk for GBS after vaccination (*21*). Data from these studies were used to communicate the safety of the influenza A(H1N1)pdm09 vaccine, thereby improving public trust and government transparency.

### Support for Policy Recommendations and Program Evaluations

The best protection against influenza and influenza-associated complications is considered to be annual influenza vaccination. In the United States since 2010–11, influenza vaccination has been recommended for all persons >6 months of age (*22*). However, since the first national influenza vaccination policy was developed in 1968 until 2010, influenza vaccination recommendations were based on risk; groups recommended to receive vaccine were added incrementally over time as evidence was produced with regard to the risk factors for and the burden of influenza among various groups. The rates of severe disease provided by this system have also been used to develop evidence for focusing on new vaccine target groups and ultimately for justifying the current universal vaccination policy (*22*).

**Figure 5:**
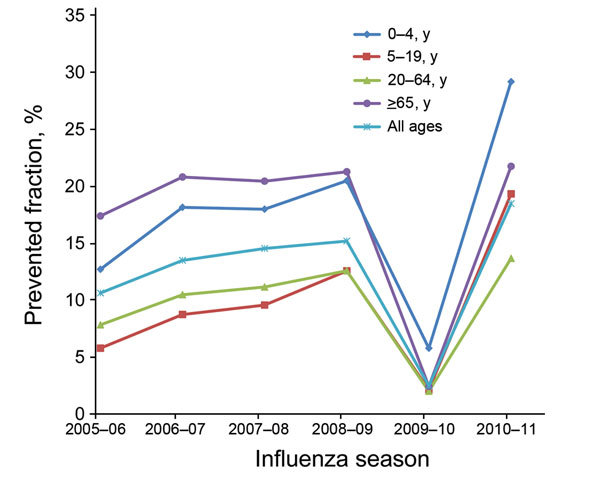
Prevented fraction of influenza cases as a result of vaccination, by age group and influenza season, United States, 2005–06 through 2010–11 influenza seasons. Prevented fraction was defined as the proportion of averted outcomes out of potential outcomes in the absence of vaccination (*15*).

In the past, the benefits of influenza vaccination have been evaluated by use of cost-effectiveness studies that assessed vaccination coverage and vaccine efficacy or effectiveness in certain groups of the population (*23*,*24*). However, data from FluSurv-NET have provided a unique mechanism for evaluating the effect of influenza vaccination on a national scale (*15*). While FluSurv-NET data have been used to develop methods for estimating disease burden (i.e., number of cases, medically attended visits, and deaths associated with influenza in the United States) (*15*,*16*), when the number of doses of vaccination administered and the effectiveness of vaccine are applied to these estimates, the effect of the vaccine program can be assessed (*15*,*25*). The continuity of the surveillance system enables estimation of vaccine impact over time, and the value of influenza vaccines can be easily communicated to stakeholders as number of influenza cases, hospitalizations, and deaths prevented each season. The data have shown that the prevented fraction of influenza cases has varied by age group and year (Figure 5). The highest estimate for a prevented fraction was from postpandemic seasons, after influenza vaccine recommendation became universal in the United States (*15*,*25*).

### Capacity to Serve as a Platform for Addressing Research Questions

Over the years, FluSurv-NET data have been used to answer various epidemiologic questions of public health relevance. Published studies have confirmed risk factors for severe clinical outcomes associated with influenza virus infection among young children who are hospitalized and those with asthma (*26*–*28*), the effect of A(H1N1)pdm09 virus infection among hospitalized pregnant women (*10*), the effect of hospital-associated influenza on clinical outcomes (*29*), and the role of alcohol abuse as a risk factor for influenza severity (*30*). Studies have explored the association between pneumonia and influenza before and during the 2009 influenza pandemic (*31*).

Another contribution from the network was a description of the association between use of statins and death among patients hospitalized with laboratory-confirmed influenza. Using FluSurv-NET data, Vandermeer et al. reported a 41% reduction in 30-day mortality rate among patients hospitalized with laboratory-confirmed seasonal influenza who were receiving statin treatment (*32*). Much discussion has involved the use of statins to improve survival rates among hospitalized patients with infectious conditions (*33*). Findings from FluSurv-NET have generated interest in further exploring use of statins to reduce severe outcomes among those with influenza virus infection. Further analyses using FluSurv-NET data to explore the potential benefit of statin treatment among hospitalized patients with influenza are under way.

Because FluSurv-NET data are geocoded, exploratory analyses looking at socioeconomic and other disparities among patients hospitalized with influenza have also been conducted (*34*,*35*). These analyses indicated a correlation between patient residence in impoverished or densely populated neighborhoods and incidence of influenza-associated hospitalization in Connecticut. Multisite analyses are under way to explore whether other patterns may be found in other states when considering various influenza seasons and age groups. Linking geocoded surveillance data and census information to identify geographic pockets of persons at higher risk for severe influenza or hospitalization may help local and state health departments prioritize targeted interventions among groups or neighborhoods at high risk for hospitalization for influenza.

Data from FluSurv-NET are also useful for describing differences in clinical severity of disease from season to season (*36*,*37*) and by type and subtype of influenza viruses (*38*,*39*). Since the 2009 influenza pandemic, more hospitals in the surveillance areas have access to molecular diagnostics, support from state public health laboratories with test confirmation and subtyping of influenza A viruses, or both. The data have enabled the exploration of severity and clinical presentation of influenza according to virus subtype during the 2010–11 season (*39*). Although the 2009 influenza pandemic was first thought of as a relatively mild pandemic, data from FluSurv-NET were able to demonstrate that infection with A(H1N1)pdm09 virus led to more severe disease in persons in all age groups, including older adults who were more likely to be admitted to an intensive care unit or require mechanical ventilation than were those infected with influenza (H3N2) or influenza B viruses, which co-circulated during the postpandemic influenza season (*39*).

## Challenges and Opportunities

Testing for influenza viruses is often underutilized because of the low sensitivity of rapid tests, lack of prompt access to RT-PCR and other molecular assays at the hospital level, and greater reliance on clinical diagnosis for influenza. As a consequence, the number of persons identified as part of influenza hospitalization surveillance is probably lower than the true number of persons hospitalized with influenza. Nonetheless, as new and more rapid molecular assays to detect influenza viruses become available in the FluSurv-NET hospitals and laboratories, testing practices for influenza may change. It is important to keep track of changes in laboratory capacity over time and to monitor testing practices at the hospital level to aid in interpretation of results and to adjust estimates of incidence rate for hospitalization.

To identify catchment area denominators for persons in high-risk groups (e.g., those with diabetes, obesity, or chronic cardiovascular disease), FluSurv-NET will also explore data from the Behavioral Risk Factor Surveillance System, a large, worldwide, ongoing telephone health survey system. This information will enable estimation of relative risk for hospitalization, intensive care unit admission, and death among persons in specific high-risk groups, facilitating outreach messaging and justifying efforts to prioritize these groups for interventions. New molecular diagnostics can identify the presence of influenza virus RNA in specimens from the respiratory tract and can discriminate between virus type and subtype in some cases. In general, these assays yield results in 1–8 hours. Moreover, multipathogen testing platforms are becoming increasingly more common in clinical settings (*40*). These new diagnostic tools can improve ascertainment of respiratory pathogens and accuracy of detection, informing clinicians of the need for additional diagnostic testing, antibacterial or antiviral therapy, and helping with decisions regarding hospitalization and infection control measures. FluSurv-NET will need to continue to monitor testing practices in its catchment area to understand the data gathered and interpret trends on influenza hospitalization and severity of seasons over time. Furthermore, the availability of reliable data on other respiratory pathogens may enable surveillance for additional causes of hospitalization for severe acute respiratory illness.

Use of metagenomics and bioinformatics can improve our understanding of the association between respiratory microbiota and the risk for severe disease associated with various respiratory pathogens, including influenza. In preparation for the role that advanced molecular detection could have in transforming existing surveillance platforms and the way surveillance data are commonly used for public health response, the FluSurv-NET platform is in a unique position to contribute respiratory specimens for sequencing and to answer questions about influenza virus evolution, antiviral drug susceptibility, molecular determinants of severity, and whether the viral molecular profiles differ among hospitalized patients with influenza who have or have not been vaccinated.

Another area of growing interest within EIP is the use of spatial epidemiology to evaluate health disparities and geographic spread of influenza. FluSurv-NET uses geocoded surveillance data linked to census tract data to look at area-based factors influencing health inequalities (e.g., poverty) instead of race/ethnicity. FluSurv-NET will use surveillance data from influenza-associated hospitalizations to assess modifiable area-based determinants of health in the community, to generate new lines of research or promote targeted interventions for persons in identified high-risk groups, and to explore the effect of differential access to care and poverty level on rates of hospitalizations. Efforts to analyze the association between different influenza seasons (by type and subtype) and socioeconomic status are under way, and collaborations with academic institutions with experience in this area are now being fostered.

## Conclusions

The EIP network was used to establish population-based surveillance for laboratory-confirmed, influenza-related hospitalizations. This system was expanded during the 2009 influenza pandemic to include additional surveillance sites and is now known as FluSurv-NET. A powerful attribute of this surveillance is that key data are collected and submitted in near real time to CDC to provide situational awareness during an influenza season. The system was enhanced during the 2009 influenza pandemic and proved to be extremely helpful for monitoring disease burden, severity, and at-risk groups. FluSurv-NET is also expanding into measuring health disparities and pathogen genomics. FluSurv-NET has proven to be a comprehensive yet efficient system for measuring severe influenza in the United States and serves as an exceptional and nimble platform for investigating questions of public health importance with regard to severe influenza.

Technical Appendix Form used to collect information on patient demographics and clinical course of illness during hospitalization for each laboratory-confirmed influenza case.
