# Supplementary material for: The US Influenza Hospitalization Surveillance Network
Source: Emerg Infect Dis. 2015 Sep;21(9):1543–50. doi: 10.3201/eid2109.141912 (PMC4550140; doi:10.3201/eid2109.141912)
Supplement: Technical Appendix — Form used to collect information on patient demographics and clinical course of illness during hospitalization for each laboratory-confirmed influenza case. [file 14-1912-Techapp-s1.pdf]

Article DOI: http://dx.doi.org/10.3201/eid2109.141912

The US Influenza Hospitalization Surveillance Network

Technical Appendix

The following pages show the form used to collect information on demographic characteristics and clinical course of illness during hospitalization for each laboratory-confirmed influenza case through review of medical records.

U.S. Department of Health and Human Services
Centers for Disease Control and Prevention

Case ID:__ __ 1 3 1 4 __ __ __ __

2013-14 FluSurv-NET Influenza Hospitalization Surveillance Project Case Report Form

A. Patient Data – THIS INFORMATION IS NOT SENT TO CDC

Last Name:	First Name:	Phone Number 1:	Phone Number 2:
Street Address:		City:	Zip:
Chart Number		Census Tract:	
Emergency Contact 1:		Emergency Contact Phone:	
Primary Provider Name:	Provider Phone Number:	Provider Fax Number:	
Site Use 1:	Site Use 2:	Site Use 3:	

B. Reporter Information – THIS INFORMATION IS NOT SENT TO CDC

1. Reporter Name: ____________________	2. Date Reported: ____/ ____/ ____

C. Enrollment Information

1. Case Classification: ☐ Prospective Surveillance ☐ Discharge Audit

2. Admission Type: ☐ Hospitalization ☐ Observation Only

3. County:

4. State:

5. Case Type: ☐ Pediatric ☐ Adult

6. Date of Birth: ____/ ____/ ____

7. Age: __________ ☐ Years ☐ Months (if < 1 yr) ☐ Days (if < 1 month)

8. Sex: ☐ Male ☐ Female

9. Race: ☐ White ☐ Black or African American ☐ Asian/Pacific Islander ☐ American Indian or Alaska Native ☐ Multiracial ☐ Not specified

10. Ethnicity: ☐ Hispanic or Latino ☐ Non-Hispanic or Latino ☐ Not Specified

11. Hospital ID Where Patient Treated: ____________ **11a. Admission Date:** _____/ _____/ _____ **11b. Discharge Date:** _____/ _____/ _____

12. Was patient transferred from another hospital? ☐ Yes ☐ No ☐ Unknown **12a. Transfer Hospital ID:** _____________

12b. Transfer Hospital Admission Date: _____/ _____/ _____ **12c. Transfer Date:** _____/ _____/ _____

13. Where did patient reside at the time of hospitalization? Indicate TYPE of residence.

☐ Private residence ☐ Rehabilitation facility ☐ Group home/Retirement home ☐ Assisted living/Residential care

☐ Homeless/Shelter ☐ Nursing home ☐ Unknown ☐ Other, specify: _____________________

13a. If resident of a facility, indicate NAME of facility: ____

D. Influenza Testing Results

1. Test 1: ☐ Rapid ☐ RT-PCR ☐ Viral Culture ☐ Serology ☐ Fluorescent Antibody ☐ Method Unknown/Note Only

1a. Result: ☐ Flu A (not subtyped) ☐ Flu B ☐ Flu A & B ☐ Flu A/B (Not Distinguished)
☐ 2009 H1N1 ☐ H1, Seasonal ☐ H1, Unspecified ☐ H3 ☐ Flu A, Unsubtypable
☐ Negative ☐ Unknown ☐ Other, specify: ________________________________

1b. Specimen collection date: ___/___/ ___ **1c. Testing facility ID:** _________________ **1d. Specimen ID:** ______________________

2. Test 2: ☐ Rapid ☐ RT-PCR ☐ Viral Culture ☐ Serology ☐ Fluorescent Antibody ☐ Method Unknown/Note Only

2a. Result: ☐ Flu A (not subtyped) ☐ Flu B ☐ Flu A & B ☐ Flu A/B (Not Distinguished)
☐ 2009 H1N1 ☐ H1, Seasonal ☐ H1, Unspecified ☐ H3 ☐ Flu A, Unsubtypable
☐ Negative ☐ Unknown ☐ Other, specify: ________________________________

2b. Specimen collection date: ___/___/ ___ **2c. Testing facility ID:** _________________ **2d. Specimen ID:** ______________________

3. Test 3: ☐ Rapid ☐ RT-PCR ☐ Viral Culture ☐ Serology ☐ Fluorescent Antibody ☐ Method Unknown/Note Only

3a. Result: ☐ Flu A (not subtyped) ☐ Flu B ☐ Flu A & B ☐ Flu A/B (Not Distinguished)
☐ 2009 H1N1 ☐ H1, Seasonal ☐ H1, Unspecified ☐ H3 ☐ Flu A, Unsubtypable
☐ Negative ☐ Unknown ☐ Other, specify: ________________________________

3b. Specimen collection date: ___/___/ ___ **3c. Testing facility ID:** _________________ **3d. Specimen ID:** ______________________

4. Test 4: ☐ Rapid ☐ RT-PCR ☐ Viral Culture ☐ Serology ☐ Fluorescent Antibody ☐ Method Unknown/Note Only

4a. Result: ☐ Flu A (not subtyped) ☐ Flu B ☐ Flu A & B ☐ Flu A/B (Not Distinguished)
☐ 2009 H1N1 ☐ H1, Seasonal ☐ H1, Unspecified ☐ H3 ☐ Flu A, Unsubtypable
☐ Negative ☐ Unknown ☐ Other, specify: ________________________________

4b. Specimen collection date: ___/___/ ___ **4c. Testing facility ID:** _________________ **4d. Specimen ID:** ______________________

E. Admission and Patient History

1. Was patient discharged from any hospital within one week prior to the current admission date? ☐ Yes ☐ No ☐ Unknown

2. Acute conditions at admission (Check all that apply):

☐ Acute respiratory illness ☐ Asthma and/or COPD exacerbation ☐ Fever ☐ Pneumonia
☐ Other respiratory or cardiac conditions ☐ Other, neither respiratory nor cardiac conditions ☐ Unknown

3. Date of onset of acute respiratory symptoms: ____/ ____/ ____ ☐ Unknown

3a. If no respiratory symptoms, date of onset of acute illness resulting in hospitalization: ____/ ____/ ____ ☐ Unknown

4. Body Mass Index: ☐ Unknown

5. Height: ☐ Inches ☐ Cm ☐ Unknown

6. Weight: ☐ Lbs ☐ Kg ☐ Unknown

7. Smoker: ☐ Current ☐ Former ☐ No/Unknown

8. Alcohol abuse: ☐ Current ☐ Former ☐ No/Unknown

9. Did patient have any of the following pre-existing medical conditions? Check all that apply. ☐ Yes ☐ No ☐ Unknown

9a Asthma/Reactive Airway Disease ☐ Yes ☐ No/Unknown

9b. Chronic Lung Disease ☐ Yes ☐ No/Unknown
- ☐ Cystic fibrosis
- ☐ Emphysema/COPD
- ☐ Other, specify____

9c. Chronic Metabolic Disease ☐ Yes ☐ No/Unknown
- ☐ Diabetes
- ☐ Thyroid dysfunction
- ☐ Other, specify____

9d. Blood disorders/Hemoglobinopathy ☐ Yes ☐ No/Unknown
- ☐ Sickle cell disease
- ☐ Splenectomy/Asplenia
- ☐ Thrombocytopenia
- ☐ Other, specify ____

9e. Cardiovascular Disease ☐ Yes ☐ No/Unknown
- ☐ Atherosclerotic cardiovascular disease (ASCVD)
- ☐ Cerebral vascular incident/Stroke
- ☐ Congenital heart disease
- ☐ Coronary artery disease (CAD)
- ☐ Heart failure/CHF
- ☐ Other, specify ____

9f. Neuromuscular disorder ☐ Yes ☐ No/Unknown
- ☐ Duchenne muscular dystrophy
- ☐ Muscular dystrophy
- ☐ Multiple sclerosis
- ☐ Mitochondrial disorder
- ☐ Myasthenia gravis
- ☐ Other, specify: ______________________________________

9g. Neurologic disorder ☐ Yes ☐ No/Unknown
- ☐ Cerebral palsy
- ☐ Cognitive dysfunction
- ☐ Dementia
- ☐ Developmental delay
- ☐ Down syndrome
- ☐ Plegias/Paralysis
- ☐ Seizure/Seizure disorder
- ☐ Other, specify: ______________________________________

9h History of Guillain-Barré Syndrome ☐ Yes ☐ No/Unknown

9i. Immunocompromised Condition ☐ Yes ☐ No/Unknown
- ☐ AIDS or CD4 count < 200
- ☐ Cancer diagnosis in last 12 months
- ☐ Complement deficiency
- ☐ HIV Infection
- ☐ Immunoglobulin deficiency
- ☐ Immunosuppressive therapy
- ☐ Organ transplant
- ☐ Stem cell transplant (e.g., bone marrow transplant)
- ☐ Steroid therapy (taken within 2 weeks of admission)
- ☐ Other, specify____

9j. Renal Disease ☐ Yes ☐ No/Unknown
- ☐ Chronic kidney disease/chronic renal insufficiency
- ☐ End stage renal disease/Dialysis
- ☐ Glomerulonephritis
- ☐ Nephrotic syndrome
- ☐ Other, specify ________________________________

9k. Other ☐ Yes ☐ No/Unknown
- ☐ Liver disease (e.g., cirrhosis, chronic hepatitis, hepatitis C)
- ☐ Morbidly obese (ADULTS ONLY)
- ☐ Obese
- ☐ Pregnant
  - ☐ If pregnant, specify gestational age in weeks: ___________
  - ☐ Unknown gestational age
- ☐ Post-partum (two weeks or less)
- ☐ Other, specify ____
____
____

9l. PEDIATRIC CASES ONLY

Abnormality of upper airway ☐ Yes ☐ No/Unknown
History of febrile seizures ☐ Yes ☐ No/Unknown
Long-term aspirin therapy ☐ Yes ☐ No/Unknown
Premature ☐ Yes ☐ No/Unknown
(gestation age < 37 weeks at birth for patients < 2yrs)
If yes, specify gestation age at birth in weeks: ________________
☐ Unknown gestational age at birth

2013-14 FluSurv-NET Influenza Hospitalization Surveillance Project Case Report Form

F. Intensive Care Unit and Interventions

1. Was the patient admitted to an intensive care unit (ICU)? ☐ Yes ☐ No ☐ Unknown
1a. Number of ICU Admissions _________ ☐ Unknown
1b. Date of first ICU Admission: ____/____/____ ☐ Unknown **1c. Date of first ICU Discharge** ____/____/____ ☐ Unknown

2. Did patient receive mechanical ventilation? ☐ Yes ☐ No ☐ Unknown

3. Did patient receive extracorporeal membrane oxygenation (ECMO or 'on bypass')? ☐ Yes ☐ No ☐ Unknown

G. Bacterial Pathogens – *Sterile or respiratory site only*

1. Were any bacterial culture tests performed with a collection date within three days of admission? ☐ Yes ☐ No ☐ Unknown

2. If yes, was there a positive culture for a bacterial pathogen? ☐ Yes ☐ No ☐ Unknown

3a. If yes, specify Pathogen 1: ____________________________________ **3b. Date of culture:** ____/____/____
3c. Site where pathogen identified: ☐ Blood ☐ Cerebrospinal fluid (CSF) ☐ Bronchoalveolar lavage (BAL) ☐ Sputum ☐ Pleural fluid ☐ Endotracheal aspirate ☐ Other, specify: ________________
3d. If Staphylococcus aureus, specify: ☐ Methicillin resistant (MRSA) ☐ Methicillin sensitive (MSSA) ☐ Sensitivity unknown
3e. If Haemophilus influenzae, specify if type B: ☐ Yes ☐ No ☐ Unknown
3f. If Neisseria meningitidis, specify serogroup: ☐ B ☐ C ☐ Y ☐ Other, specify: ____________ ☐ Unknown

4a. Specify Pathogen 2: ____________________________________ **4b. Date of culture:** ____/____/____
4c. Site where pathogen identified: ☐ Blood ☐ Cerebrospinal fluid (CSF) ☐ Bronchoalveolar lavage (BAL) ☐ Sputum ☐ Pleural fluid ☐ Endotracheal aspirate ☐ Other, specify: ____________________
4d. If Staphylococcus aureus, specify: ☐ Methicillin resistant (MRSA) ☐ Methicillin sensitive (MSSA) ☐ Sensitivity unknown
4e. If Haemophilus influenzae, specify if type B: ☐ Yes ☐ No ☐ Unknown
4f. If Neisseria meningitidis, specify serogroup: ☐ B ☐ C ☐ Y ☐ Other, specify: ____________ ☐ Unknown

H. Viral Pathogens

1. Was patient tested for any of the following viral respiratory pathogens *within 3 days* of admission? ☐ Yes ☐ No ☐ Unknown

1a. Respiratory syncytial virus/RSV	☐ Yes, positive	☐ Yes, negative	☐ Not tested/Unknown	**Date**: ____/____/____
1b. Adenovirus	☐ Yes, positive	☐ Yes, negative	☐ Not tested/Unknown	**Date**: ____/____/____
1c. Parainfluenza 1	☐ Yes, positive	☐ Yes, negative	☐ Not tested/Unknown	**Date**: ____/____/____
1d. Parainfluenza 2	☐ Yes, positive	☐ Yes, negative	☐ Not tested/Unknown	**Date**: ____/____/____
1e. Parainfluenza 3	☐ Yes, positive	☐ Yes, negative	☐ Not tested/Unknown	**Date**: ____/____/____
1f. Human metapneumovirus	☐ Yes, positive	☐ Yes, negative	☐ Not tested/Unknown	**Date**: ____/____/____
1g. Rhinovirus	☐ Yes, positive	☐ Yes, negative	☐ Not tested/Unknown	**Date**: ____/____/____
1h. Other, specify: ____________	☐ Yes, positive	☐ Yes, negative	☐ Not tested/Unknown	**Date**: ____/____/____

I. Influenza Treatment

1. Did patient receive antiviral medication treatment for influenza during the course of this illness? ☐ Yes ☐ No ☐ Unknown

2a. Treatment 1: ☐ Oseltamivir (Tamiflu) ☐ Zanamivir (Relenza) ☐ Other, specify: ____________________________
☐ Amantadine (Symmetrel) ☐ Rimantadine (Flumadine) ☐ Unknown
2b. Method of Administration: ☐ Oral ☐ Intravenous (IV) ☐ Inhaled ☐ Unknown
2c. Start Date: ___/____/____ **2d. End Date:** ____/____/____ **2e. Dose** ________________ **2f. Frequency**: ________________
☐ Start Date Unknown ☐ End Date Unknown ☐ Dose Unknown ☐ Frequency Unknown

3a. Treatment 2: ☐ Oseltamivir (Tamiflu) ☐ Zanamivir (Relenza) ☐ Other, specify: ____________________________
☐ Amantadine (Symmetrel) ☐ Rimantadine (Flumadine) ☐ Unknown
3b. Method of Administration: ☐ Oral ☐ Intravenous (IV) ☐ Inhaled ☐ Unknown
3c. Start Date: ___/____/____ **3d. End Date:** ____/____/____ **3e. Dose** ________________ **3f. Frequency**: ________________
☐ Start Date Unknown ☐ End Date Unknown ☐ Dose Unknown ☐ Frequency Unknown

4a. Treatment 3: ☐ Oseltamivir (Tamiflu) ☐ Zanamivir (Relenza) ☐ Other, specify: ____________________________
☐ Amantadine (Symmetrel) ☐ Rimantadine (Flumadine) ☐ Unknown
4b. Method of Administration: ☐ Oral ☐ Intravenous (IV) ☐ Inhaled ☐ Unknown
4c. Start Date: ___/____/____ **4d. End Date:** ____/____/____ **4e. Dose** ________________ **4f. Frequency**: ________________
☐ Start Date Unknown ☐ End Date Unknown ☐ Dose Unknown ☐ Frequency Unknown

5a. Treatment 4: ☐ Oseltamivir (Tamiflu) ☐ Zanamivir (Relenza) ☐ Other, specify: ____________________________
☐ Amantadine (Symmetrel) ☐ Rimantadine (Flumadine) ☐ Unknown
5b. Method of Administration: ☐ Oral ☐ Intravenous (IV) ☐ Inhaled ☐ Unknown
5c. Start Date: ___/____/____ **5d. End Date:** ____/____/____ **5e. Dose** ________________ **5f. Frequency**: ________________
☐ Start Date Unknown ☐ End Date Unknown ☐ Dose Unknown ☐ Frequency Unknown

6. Additional Treatment Comments:

2013-14 FluSurv-NET Influenza Hospitalization Surveillance Project Case Report Form

J. Chest Radiograph – *Based on radiology report only*

1. Was a chest x-ray taken *within 3 days* of admission? ☐ Yes ☐ No ☐ Unknown

2. Were any of these chest x-rays abnormal? ☐ Yes ☐ No ☐ Unknown

2a. Date of first abnormal chest x-ray: ____/____/____

2b. For first abnormal chest x-ray, please check all that apply:

☐ Report not available ☐ Consolidation ☐ Interstitial infiltrate
☐ Air space density/opacity ☐ Atelectasis ☐ Pleural effusion/empyema
☐ Bronchopneumonia/pneumonia ☐ Cavitation ☐ Lobar (NOT interstitial) infiltrate
☐ Cannot rule out pneumonia ☐ ARDS (acute respiratory distress syndrome) ☐ Other

2c. Please specify location for bronchopneumonia/pneumonia/consolidation/lobar infiltrate/air space density/opacity:

☐ Single lobar ☐ Multiple lobar (unilateral) ☐ Multiple lobar (bilateral) ☐ Unknown

K. Discharge Summary

1. Did the patient have any of the following diagnoses at discharge (check all that apply)?

Pneumonia	☐ Yes	☐ No	☐ Unknown	Stroke (CVI)	☐ Yes	☐ No	☐ Unknown
Guillain-Barré syndrome	☐ Yes	☐ No	☐ Unknown	Acute myocarditis	☐ Yes	☐ No	☐ Unknown
Acute encephalopathy/ encephalitis	☐ Yes	☐ No	☐ Unknown	Acute respiratory distress syndrome (ARDS)	☐ Yes	☐ No	☐ Unknown
Seizures	☐ Yes	☐ No	☐ Unknown	Bronchiolitis	☐ Yes	☐ No	☐ Unknown
Reye's syndrome	☐ Yes	☐ No	☐ Unknown	Hemophagocytic syndrome	☐ Yes	☐ No	☐ Unknown

2. What was the outcome of the patient? ☐ Alive ☐ Deceased ☐ Unknown

2a. If discharged alive, please indicate to where:

☐ Home ☐ Other hospital ☐ Hospice/Home hospice ☐ Homeless/Shelter
☐ Rehabilitation Facility ☐ Group home/Retirement home ☐ Assisted living/Residential Care ☐ Unknown
☐ Home with Services ☐ Nursing home ☐ Other, specify: ____________________

3. If patient was pregnant on admission, indicate pregnancy status at discharge: ☐ Still pregnant ☐ No longer pregnant ☐ Unknown

3a. If patient was pregnant on admission but no longer pregnant at discharge, indicate pregnancy outcome at discharge:

☐ Miscarriage ☐ Ill newborn ☐ Newborn died ☐ Healthy newborn ☐ Abortion ☐ Unknown

4. Additional notes regarding discharge: ____

L. ICD-9 or ICD-10 Discharge Diagnoses – *To be recorded in order of appearance*

**Version:** ☐ ICD-9 ☐ ICD-10	1.	4.	7.
	2.	5.	8.
	3.	6.	9.

M. Vaccination History

1. Did patient receive the influenza vaccine for the current influenza season? ☐ Yes ☐ No ☐ Unknown

Specify vaccination status and date(s) by source:

2. Medical Chart ☐ Yes ☐ Yes, specific date unknown ☐ No ☐ Unknown ☐ Not Checked
2a. If yes, specify dosage date information: 1) ___/___/___ ☐ Date Unknown 2) (Pediatrics Only) ___/___/___ ☐ Date Unknown

3.Vaccine Registry ☐ Yes ☐ Yes, specific date unknown ☐ No ☐ Unknown ☐ Not Checked
3a. If yes, specify dosage date information: 1) ___/___/___ ☐ Date Unknown 2) (Pediatrics Only) ___/___/___ ☐ Date Unknown

4. Primary Care Provider / Long-term Care Facility ☐ Yes ☐ Yes, specific date unknown ☐ No ☐ Unknown ☐ Not Checked
4a. If yes, specify dosage date information: 1) ___/___/___ ☐ Date Unknown 2) (Pediatrics Only) ___/___/___ ☐ Date Unknown

5. Interview: ☐ Patient ☐ Proxy ☐ Yes ☐ Yes, specific date unknown ☐ No ☐ Unknown ☐ Not Checked
5a. If yes, specify dosage date information: 1) ___/___/___ ☐ Date Unknown 2) (Pediatrics Only) ___/___/___ ☐ Date Unknown

6. Other, specify: ____________________ ☐ Yes ☐ Yes, specific date unknown ☐ No ☐ Unknown ☐ Not Checked
6a. If yes, specify dosage date information: 1) ___/___/___ ☐ Date Unknown 2) (Pediatrics Only) ___/___/___ ☐ Date Unknown

7. If patient < 9 years, did patient receive any seasonal influenza vaccine in previous seasons? ☐ Yes ☐ No ☐ Unknown

N. Miscellaneous

1. Case Finding: ☐ Hospital Log ☐ Laboratory List ☐ Discharge Database ☐ Reportable Disease ☐ Other, specify: __________

2. Additional Comments:
